# Breast cancer radiotherapy: analysis of unintended internal mammary node doses and influencing factors

**DOI:** 10.1590/1806-9282.20241325

**Published:** 2025-03-31

**Authors:** Fevziye İlknur Kayalı, Rahşan Habiboğlu, İpek Pınar Aral, Volkan Çevik, Yılmaz Tezcan

**Affiliations:** 1 Ankara Bilkent City Hospital, Department of Radiation Oncology – Ankara, Turkey.; 2 Ankara Yıldırım Beyazıt University, Ankara Bilkent City Hospital, Radiation Oncology Clinic, Department of Radiation Oncology – Ankara, Turkey.

**Keywords:** Breast cancer, Radiotherapy, Dosage, Internal mammary

## Abstract

**OBJECTIVE:**

Breast cancer is a prevalent malignancy requiring ongoing treatment advancements. Radiotherapy is vital for reducing recurrence and improving survival. This study evaluates unintended doses to internal mammary lymph nodes and influencing factors in patients at Ankara Bilkent City Hospital's Radiation Oncology Clinic.

**METHODS:**

We analyzed 44 right-sided breast cancer patients treated with radiotherapy between November 2019 and April 2023. Data on demographics, treatment, and dose–volume histograms were reviewed using various statistical tests.

**RESULTS:**

Median age was 54 years; 88.6% had invasive ductal carcinoma, and 11.4% had ductal carcinoma in situ. Patients received conventional (54.5%) or hypofractionated radiotherapy (45.5%) using intensity-modulated radiotherapy or three-dimensional conformal radiotherapy. Median internal mammary lymph node volume was 7.3 cc with dose variability. Internal mammary lymph nodes V45 dose showed no correlation with internal mammary lymph nodes volume, radiotherapy field, pT stage, or pN stage. However, the nodal stage significantly impacted the internal mammary lymph nodes D95 dose, with higher doses in N1 patients. Wider radiotherapy fields led to increased D95 doses.

**DISCUSSION:**

The findings highlight the variability in internal mammary lymph nodes doses and the impact of nodal stage and radiotherapy field on dose distribution. Advanced techniques like intensity-modulated radiotherapy can reduce risks, but careful planning is essential.

**CONCLUSION:**

Understanding internal mammary lymph nodes dose factors can enhance treatment planning and outcomes. Future research should focus on refining guidelines and leveraging technology to improve radiotherapy efficacy.

## INTRODUCTION

Breast cancer remains one of the most prevalent malignancies worldwide, necessitating continuous advancements in treatment strategies to improve patient outcomes. Among the multifaceted approaches to breast cancer management, radiotherapy (RT) plays a crucial role, particularly in reducing local recurrence and improving overall survival rates. The incorporation of regional nodal irradiation (RNI) and internal mammary lymph node irradiation (IMNI) has been a focal point of research, aiming to enhance the efficacy of RT while minimizing associated risks. The early breast cancer trialists’ collaborative group meta-analysis demonstrated that post-mastectomy RT significantly reduces locoregional recurrence, overall recurrence, and breast cancer mortality in women with node-positive disease^1^. These findings underscore the importance of optimized local therapy in the era of molecular subtype-guided adjuvant systemic therapy. Similarly, the MA.20 trial highlighted the benefits of adding RNI, including IMNI, to whole breast irradiation, showing significant improvements in disease-free survival and distant disease-free survival in node-positive and high-risk node-negative breast cancer patients^2,3^. Despite the evident benefits, the role of IMNI has been a subject of debate due to the potential increase in cardiac and pulmonary irradiation dose–volume. The risk of secondary malignancies and ischemic heart disease with traditional RT techniques has necessitated the exploration of advanced modalities such as intensity-modulated radiotherapy (IMRT) and three-dimensional conformal radiotherapy (3D-CRT)^4,5^. Studies by Overgaard et al. have shown that while IMNI enhances RT outcomes, it also significantly increases the dose exposure to critical organs, thereby elevating the risks of secondary lung cancer, contralateral breast cancer, and ischemic heart disease^6^. However, IMRT has been found to reduce these risks compared to 3D-CRT, highlighting the need for a balanced approach in clinical practice^6^.

The primary objective of this study is to analyze the unintended doses delivered to the internal mammary lymph nodes (IMNs) in breast cancer RT and identify the parameters influencing these doses. The importance of understanding these dose factors lies in enhancing treatment safety and improving patient outcomes. While RNI, which includes IMN, has shown to improve survival outcomes, the role of incidental doses to the IMN and their impact on both therapeutic benefits and potential toxicities remain debated. Thus, this study aims to investigate factors affecting unintended IMN doses in an effort to contribute to safer, more effective RT strategies.

## METHODS

### Study design

In this study, a retrospective analysis was performed on 44 patients diagnosed with breast cancer who received RT at the Radiation Oncology Clinic of Ankara Bilkent City Hospital between November 2019 and April 2023. Patient information, patient files, dose–volume histograms, and electronic system data were used for the study. Demographic data, presenting symptoms, radiological and pathological analysis details, treatments administered, and the current status of the patients were recorded. Staging was conducted according to AJCC version 8.

### Patient selection

Patients included in the study were those with pathologically confirmed breast cancer who received curative RT at the Radiation Oncology Clinic of Ankara Bilkent City Hospital, had complete file data, and had right-sided localized disease with an ECOG performance status of 0–3. Patients with left-sided breast cancer, metastatic disease, or without pathological confirmation were excluded from the study.

The inclusion of only patients with right-sided breast cancer in this study is justified by the use of the deep inspiration breath hold technique in left-sided breast cancer. This technique is highly dependent on patient compliance, which can complicate the creation of a homogeneous patient group. Since the focus of our analysis was on the doses delivered to the IMNs, right-sided breast cancer patients were included in the study. This approach enhances the comparability of the obtained data, allowing for clearer and more reliable results.

### Primary endpoint

The primary endpoint of the study was the analysis of IMN doses and the evaluation of parameters affecting IMN doses. The IMN was contoured from ribs 1–5, and a 5-mm margin was added to define the PTV IMN.

### Statistical analysis

Data were recorded using SPSS version 26 (IBM Corp, Armonk, NY, USA). Descriptive statistics for continuous variables included mean, standard deviation, minimum, and maximum values, while categorical variables were expressed as counts (n) and percentages (%). The normality of distribution was assessed using the Kolmogorov-Smirnov and Shapiro-Wilk tests, and nonparametric tests were used since the data did not follow a normal distribution. Chi-square and Fisher's exact tests were used to calculate the categorical demographic characteristics of the patients. Spearman's rank correlation test was used for univariate correlation analysis. The Mann-Whitney U test was employed for binary independent group analysis, while the Kruskal-Wallis test was used for the analysis of three or more independent groups, followed by post hoc analysis with the Mann-Whitney U test. A p-value of 0.05 or less was considered statistically significant.

### Ethical considerations

The study was conducted in accordance with the Declaration of Helsinki and was approved by the Ethics Committee of Ankara City Hospital on 22/03/2023, with approval number E1-23-3377.

## RESULTS

In our study, the outcomes of 44 patients diagnosed with right-sided breast cancer who received RT at the Radiation Oncology Clinic of Ankara Bilkent City Hospital between November 2019 and April 2023 were analyzed. At the time of RT initiation, the median age of the patients was 54 years (range 25–78). Pathological evaluation revealed that 39 patients (88.6%) had invasive ductal carcinoma, while 5 patients (11.4%) had ductal carcinoma in situ (DCIS). The median total RT dose administered was 50 Gy (range 50–60 Gy). Among these patients, 24 (54.5%) received conventional RT and 20 (45.5%) received hypofractionated RT. The RT techniques employed included IMRT in 17 patients (38.6%) and 3D-CRT in 27 patients (61.4%). Patient and treatment details are summarized in Table 1.

**Table 1:** Patient and treatment details.

Variable	Value
Age	Median (range): 54 (25–78)
Pathology	Invasive ductal carcinoma: 39 (88.6%)
	DCIS: 5 (11.4%)
pT stage	pT0: 2 (4.5%)
	pT1: 17 (38.6%)
	pT2: 19 (43.2%)
	pT4: 1 (2.3%)
	Tis: 5 (11.4%)
pN stage	N0: 25 (56.8%)
	N1: 17 (38.6%)
	N2: 1 (2.3%)
	N3: 1 (2.3%)
RT	Conventional: 24 (54.5%)
	Hypofractionated: 20 (45.5%)
RT technique	IMRT: 17 (38.6%)
	3D-CRT: 27 (61.4%)
RT total dose	50 Gy: 20 (45.5%)
	60 Gy: 24 (54.5%)
RT site	Breast: 23 (52.3%)
	Breast+axilla+SCF+IMN: 21 (47.7%)

DCIS: ductal carcinoma in situ; RT: radiotherapy; IMRT: intensity-modulated radiation therapy; 3D-CRT: three-dimensional conformal radiotherapy; SCF: supraclavicular fossa; IMN: internal mammary nodes.

### Detailed analysis of internal mammary lymph node dose

The median IMN volume among the patients was 7.3 cc (range 4.4–105 cc). Details of the IMN dose parameters are summarized in Table 2.

**Table 2:** Internal mammary lymph node dose details.

Parameter	Median (range)	p-value	z-value
Volume (cc)	7.3 (4.4–105)	–	–
Minimum dose (Gy)	0.061 (0.002–28.521)	0.210	–
Maximum dose (Gy)	1.029 Gy (0.039–52.883)	0.013	-2,497
Mean dose (Gy)	0.518 Gy (0.011–47.373)	0.037	-2,087
V45 (%)	5 (0–93.7)	0.595	–
D95 (Gy)	5.36 (1.42–44.35)	0.197	–

There was no significant correlation between the IMN V45 dose (%) and IMN volume (p=0.480), RT field (p=0.951), pT stage (p=0.107), or pN stage (p=0.695). Similarly, there was no significant correlation between the IMN D95 dose (Gy) and IMN volume (p=0.169), RT technique (p=0.197), or pT stage (p=0.303). However, a significant relationship was observed between the nodal stage and IMN D95 dose (p<0.02, Z=-3,524) (Table 3). Given that there were only single cases for N2 and N3 stages, comparisons were made between N0 and N1 stages, with higher D95 doses observed in N1 patients. Additionally, patients treated with RT fields including only the breast exhibited significantly lower D95 doses compared to those with fields encompassing the breast, axilla, Supraclavicular Fossa (SCF), and IMN (p<0.001, Z=-3,653) (Table 3).

**Table 3:** Relationship between nodal stage, radiotherapy site, and D95 dose.

Category		D95 (Gy) median (range)	p-value	z-value
Nodal stage				
	N0	3.47 (1.42–12.48)	<0.02	-3,524
	N1	8.29 (3.65–44.35)	–	–
	N2	13.19	–	–
	N3	5.19	–	–
RT site				
	Breast	3.07 (1.42–8.80)	<0.001	–3,653
	Breast+axilla+SCF+IMN	6.63 (3.65–44.35)	–	–

RT: radiotherapy; SCF: supraclavicular fossa; IMN: internal mammary nodes.

## DISCUSSION

In our study, we evaluated the incidental or unintended doses of the IMN, and the parameters affecting these doses of 44 patients diagnosed with right-sided breast cancer who underwent RT at the Radiation Oncology Clinic of Ankara Bilkent City Hospital between November 2019 and April 2023. The median age of our patients was 54 (range 25–78) years. This age range is consistent with the typical age range of patients undergoing RT for breast cancer treatment^7^. Similar age groups have been reported in other studies as well^8^. Pathological evaluation revealed that 88.6% of patients had invasive ductal carcinoma, while 11.4% had DCIS. It is reported in the literature that the majority of breast cancer cases are invasive ductal carcinoma, which is consistent with our findings^9^. In our study, 54.5% of patients received conventional RT, while 45.5% received hypofractionated RT. In the literature, hypofractionated RT has been shown to be effective and safe in breast cancer treatment^10^. Hypofractionated RT has been reported to improve the quality of life and treatment outcomes in breast cancer patients^11^. Additionally, 38.6% of patients received IMRT, while 61.4% received 3D-CRT. Despite the advantages of dose distribution provided by IMRT, 3D-CRT is widely used^12^. The inclusion of patients treated with 3D-CRT in our study is grounded in clinical relevance, despite the growing preference for techniques like IMRT and volumetric-modulated arc therapy ^13^. Research indicates that while IMRT significantly improves plan conformity and reduces doses to the ipsilateral lung and heart, 3D-CRT remains advantageous for minimizing low-dose volume exposure^14^. Furthermore, our findings showed no significant correlation between the IMN D95 dose (Gy) and IMN volume (p=0.169), RT technique (p=0.197), or pT stage (p=0.303), suggesting that 3D-CRT may still effectively deliver the desired doses to IMNs in selected patient populations^15,16^. In this context, evaluating the outcomes of 3D-CRT allows for a comprehensive understanding of treatment efficacy, especially as patient characteristics and treatment goals evolve.

A detailed analysis of IMN dose parameters was performed in our study. The median IMN volume was found to be 7.3 cc (range 4.4–105 cc). This volume measurement is consistent with previous studies, which have reported a wide variation in IMN volume among breast cancer patients^17^.

In terms of dose parameters, the median minimum dose delivered to the IMN was 0.061 Gy (range 0.002–28.521 Gy), the median maximum dose was 1.029 Gy (range 0.039–52.883 Gy), and the median mean dose was 0.518 Gy (range 0.011–47.373 Gy). These findings highlight the variability in dose distribution to the IMN among patients undergoing breast cancer RT.

Further analysis revealed that there was no significant correlation between the IMN V45 dose (%) and IMN volume, RT field, pT stage, or pN stage. Similarly, there was no significant correlation between the IMN D95 dose (Gy) and IMN volume, RT technique, or pT stage. However, a significant relationship was observed between the nodal stage and IMN D95 dose, with higher doses observed in N1 patients compared to N0 patients. This suggests that the nodal stage may influence the dose delivered to the IMN during RT. Additionally, patients treated with RT fields including only the breast exhibited significantly lower D95 doses compared to those with fields encompassing the breast, axilla, SCF, and IMN. This underscores the importance of considering the treatment field in assessing IMN dose parameters and optimizing RT planning to minimize unintended irradiation of the IMN.

It has been emphasized that IMN doses are closely associated with treatment efficacy and toxicity^18^. When evaluating the relationships between IMN dose parameters and clinical and treatment variables, no significant relationship was found between IMN V45 dose and IMN volume, RT field, pT, and pN stages. However, a significant relationship was observed between the IMN D95 dose and nodal stage (p<0.02), with higher D95 doses identified in N1 stage patients. This finding supports the impact of the nodal stage on treatment outcomes in the literature^19^. The pivotal role of the nodal stage in dose distribution and treatment outcomes has been emphasized^7^.

In line with the findings of our study, recent research has further illuminated the complexities of breast cancer treatment. A cross-sectional study published in Clinics highlights the importance of comprehensive diagnosis methods and molecular subtypes in advanced-stage breast cancer. This underscores the necessity for tailored RT approaches, taking into account individual patient characteristics^20^. Additionally, the study on locally advanced breast cancer emphasizes the role of breast-conserving surgery and other factors linked to overall survival after neoadjuvant treatment, reinforcing the idea that surgical and RT techniques should be strategically integrated to optimize outcomes^21^. Furthermore, a retrospective cohort study focusing on young breast cancer patients in Brazil provides insight into the demographic influences on prognosis, indicating that age and other sociodemographic factors must be considered when evaluating treatment efficacy^22^. These findings align with our observation of significant variability in IMN doses and their potential impact on treatment outcomes.

Significant differences were found between only breast RT field and areas including breast, axilla, SCF, and IMN (p<0.001). It was observed that wider RT fields led to higher D95 doses. This finding is consistent with the literature suggesting that wider areas may result in higher radiation doses and increased toxicity risks^8^. Moreover, a study investigating the measurement of extracapsular extension in sentinel lymph nodes highlights the potential predictive value of such factors for residual axillary disease, indicating that understanding lymph node involvement is crucial for effective treatment planning^23^. It has been stated that wider RT fields may lead to higher doses and increased toxicity^9^. The side effects and toxicity rates of RT have been widely discussed in the literature. Bentzen and colleagues^7^ emphasized the need for careful monitoring of early and late radiation toxicity. It should be considered, especially for RT applied to wider areas, that toxicity may increase^1^. The study by Darby et al. demonstrated an increased risk of ischemic heart disease after breast cancer RT^1^. Therefore, it was concluded that the dose should be carefully controlled, especially in treatment areas involving critical structures such as IMN.

## CONCLUSION

The data obtained in this study provide important information to understand the effects of different RT techniques and incidental dose distributions of the IMNs, and the parameters affecting these doses in right-sided breast cancer treatment. Our findings are largely consistent with the reported data in the literature, demonstrating that wider RT fields may lead to higher doses and the nodal stage is a determining factor in IMN doses.

In conclusion, the results of this study contribute important data on the dosimetric distribution to IMN and suggest that nodal stage and treatment field configurations should be carefully considered in treatment planning. Future research should aim to refine guidelines further, utilizing emerging evidence and advances in radiation therapy technology to enhance the safety and efficacy of breast cancer RT.
